# Lenalidomide in combination with dexamethasone at first relapse in comparison with its use as later salvage therapy in relapsed or refractory multiple myeloma

**DOI:** 10.1111/j.1600-0609.2009.01257.x

**Published:** 2009-06

**Authors:** Edward A Stadtmauer, Donna M Weber, Ruben Niesvizky, Andrew Belch, Miles H Prince, Jesús F San Miguel, Thierry Facon, Marta Olesnyckyj, Zhinuan Yu, Jerome B Zeldis, Robert D Knight, Meletios A Dimopoulos

**Affiliations:** 1 Abramson Cancer Center, University of PennsylvaniaPhiladelphia, PA, USA; 2 M. D. Anderson Cancer CenterHouston, TX, USA; 3 Weill Cornell Medical CollegeNew York, NY, USA; 4 Cross Cancer InstituteEdmonton, Alberta, Canada; 5 Peter MacCallum Cancer Institute Division of Haematology/Medical Oncology and University of MelbourneMelbourne, Australia; 6 Servicio de Hematología, Hospital Universitario de Salamanca, CIC, IBMCC (USAL-CSIC)Salamanca, Spain; 7 Hôpital Claude HuriezLille, France; 8 Celgene CorporationSummit, NJ, USA; 9 University of Athens School of MedicineAthens, Greece

**Keywords:** lenalidomide, multiple myeloma, relapse, time to progression, survival

## Abstract

This subset analysis of data from two phase III studies in patients with relapsed or refractory multiple myeloma (MM) evaluated the benefit of initiating lenalidomide plus dexamethasone at first relapse. Multivariate analysis showed that fewer prior therapies, along with β_2_-microglobulin (≤2.5 mg/L), predicted a better time to progression (TTP; study end-point) with lenalidomide plus dexamethasone treatment. Patients with one prior therapy showed a significant improvement in benefit after first relapse compared with those who received two or more therapies. Patients with one prior therapy had significantly prolonged median TTP (17.1 vs. 10.6 months; *P*=0.026) and progression-free survival (14.1 vs. 9.5 months, *P*=0.047) compared with patients treated in later lines. Overall response rates were higher (66.9% vs. 56.8%, *P*=0.06), and the complete response plus very good partial response rate was significantly higher in first relapse (39.8% vs. 27.7%, *P*=0.025). Importantly, overall survival was significantly prolonged for patients treated with lenalidomide plus dexamethasone with one prior therapy, compared with patients treated later in salvage (median of 42.0 vs. 35.8 months, *P*=0.041), with no differences in toxicity, dose reductions, or discontinuations despite longer treatment. Therefore, lenalidomide plus dexamethasone is both effective and tolerable for second-line MM therapy and the data suggest that the greatest benefit occurs with earlier use.

Patients with multiple myeloma (MM) will relapse despite treatment with conventional chemotherapy or high-dose chemotherapy with autologous stem cell transplantation (ASCT) (1). Newer agents in MM such as thalidomide, bortezomib, and lenalidomide have improved survival rates of patients with this disease. Combinations of these new agents have shown response rates superior to standard chemotherapy and rivalling ASCT (2–11). Nevertheless, the best treatment regimen, combinations or sequence of therapy for patients with this incurable disease in the second-line setting have yet to be standardized.

Lenalidomide is an oral immunomodulatory drug with demonstrated efficacy and tolerability in MM (2–5). It has been approved for clinical use in the USA, Europe, and Canada for previously treated MM based on the results from two phase III trials – MM-009 and MM-010. These trials assessed patients in relapsed or refractory disease with a median of two and three prior therapies, respectively (2, 3).

The aim of this subset analysis of the MM-009 and MM-010 studies was to evaluate the benefit of lenalidomide plus dexamethasone after only one prior therapy and compare outcomes for patients treated in later salvage therapy. This is the first such analysis, and these data will provide a valuable insight when considering the overall treatment plan and therapy sequence for patients.

## Methods

### Study design

Patients treated with lenalidomide plus dexamethasone enrolled in the two large, randomized, multicentre clinical trials of lenalidomide plus dexamethasone vs. dexamethasone alone [MM-009 (2) and MM-010 (3)] were included in this preplanned analysis. In the MM-009 and MM-010 studies, patients with MM who had received at least one prior treatment, and were not resistant to dexamethasone, were randomized to receive either oral lenalidomide (25 mg daily for 21 d of every 28-d cycle) plus dexamethasone (40 mg on day 1–4, 9–12, and 17–20 every 28 d for 4 months, then 40 mg on day 1–4 every cycle thereafter until disease progression or treatment intolerance), or dexamethasone (same dose and schedule as above) plus placebo. Patients were stratified at randomization by investigator-reported baseline β_2_-microglobulin (≤ 2.5 mg/L vs. >2.5 mg/L), prior therapies (whether or not the patient had undergone prior treatment with high-dose chemotherapy, and stem cell transplantation) and number of prior therapies (1 vs. >1).

For this analysis, determination of number of prior lines of antimyeloma therapy was retrospectively assessed in order to derive a uniform definition of line of therapy:

1. Induction chemotherapy for peripheral-blood stem cell harvest followed by planned mobilization and subsequent high-dose chemotherapy with ASCT was considered one therapy regardless of the induction regimen.
2. If the first treatment was followed by maintenance with interferon or steroids, then it was still considered as one line of therapy. Maintenance therapy was not considered a separate line of therapy.
3. Two ASCTs within 6 months of each other was considered as one line unless different agents were used in the high-dose therapy-conditioning regimens.
4. If the same regimen was repeated after a 6-month interval, they were considered to be two separate therapeutic lines.
5. If cyclophosphamide was used for reasons other than planned stem cell mobilization, its use was considered to be a separate line of therapy.
6. If dexamethasone was used once, it did not count as a line of therapy.
7. If a regimen was stopped for more than 2 months, its reinitiation was counted as another line of therapy.

The international uniform response criteria for MM were used to evaluate response (12) and the European Group for Blood and Marrow Transplant response criteria for MM (13) were used to evaluate time to progression (TTP) and progression-free survival (PFS) per protocol. TTP was measured from randomization to the date of the first assessment showing disease progression. Patients who died or discontinued the study without evidence of disease progression were censored at the last evaluation for assessment of TTP. PFS was measured from randomization to the date of the first assessment showing disease progression, or death during treatment. Patients who were alive and discontinued the study without evidence of disease progression were censored at the last evaluation for assessment of PFS. Overall survival (OS) was calculated as the time from randomization until death from any cause, or censored at the last follow-up visit. Overall response rate (ORR), TTP, PFS, and OS were all evaluated as measures of clinical benefit. Toxic effects were graded according to the National Cancer Institute Common Toxicity Criteria (NCI-CTC), version 2.

### Statistical analysis

The MM-009 and MM-010 trials were initiated on 27 February 2003, and 22 September 2003, respectively. Unblinding occurred in June and August 2005, respectively, due to demonstrated superiority of lenalidomide plus dexamethasone over dexamethasone alone at the interim analysis and upon recommendation of the studies’ independent data-monitoring committee. Data on ORR, TTP, PFS, and OS were assessed up to these dates, with a median follow-up duration of 17.6 months (range: 11.0–25.6) for active patients. Follow-up data on OS have been updated and were obtained up to 11 December 2008, and the median follow-up duration for surviving patients was 51 months (range: 0.6–66.5).

Analyses of patients treated with lenalidomide plus dexamethasone (*n*=353) were conducted using sas version 9.1 [Lenalidomide (Revlimid®), Celgene Corporation, Summit, NJ, USA]. The Cox proportional hazards model was first performed as an exploratory analysis to determine which demographic and prognostic variables most affected the primary efficacy end-point, TTP. Only those variables that differed from a preliminary analysis at the 0.20 level were included in the final multivariate model. A forward-selection stepwise procedure was used to identify the subset of relevant factors.

The final model showed that the number of prior therapies was one of the only two significant predictors for TTP, after controlling for other baseline variables. Therefore, further analysis was conducted using a univariate model to examine the efficacy outcome of the patient subgroups in the combination arm, with one prior therapy vs. those with at least two prior therapies.

Kaplan–Meier methods were used to estimate time-to-event variables with censoring, including TTP, PFS, OS, and response duration. Two-sided log rank tests were used to compare survivorship functions between treatment groups for these time-to-event end-points. Fisher’s exact tests were used to compare response rates. All of the analyses were performed without prespecified power calculation or adjustment for multiplicity, and are therefore considered exploratory in nature.

## Results

### Multivariate analysis

Variables assessed in the preliminary multivariate Cox regression model included the following baseline characteristics: gender, age, race, percentage of plasma cells in the bone marrow, time since diagnosis, baseline Eastern Cooperative Oncology Group performance status, baseline disease stage, presence of worsening lytic bone lesions at baseline, β_2_-microglobulin, prior thalidomide, prior bortezomib, prior radiation therapy, duration of MM, whether there was presence of baseline bone lesions, and the number of prior antimyeloma therapies, including stem cell transplantation. Only those variables that differed from the preliminary analysis at the 0.20 level were included in the final multivariate model. The final model showed that number of prior therapies was one of the only two significant baseline prognostic factors that predicted TTP for patients treated with lenalidomide plus dexamethasone (Table 1). In other words, the fewer prior therapies a patient had, the less likely it is that the disease will progress and the longer the TTP will be. Use of prior thalidomide or bortezomib did not impact the TTP outcome (i.e. these two variables were not included in the final model).

**Table 1:** Multivariate analysis (*n*=353)

Variable	HR	95% CI	*P*-value
β_2_-microglobulin (>2.5 mg/L vs. ≤2.5 mg/L)	1.622	1.280–2.056	<0.0001
Number of prior antimyeloma therapies	1.181	1.056–1.321	0.0032
Duration of multiple myeloma (yr)	0.964	0.928–1.002	0.0614
Age (yr)	0.991	0.981–1.002	0.0958

CI, confidence interval; HR, hazard ratio.

Given that the number of prior therapies was a significant predictor of TTP, further analysis was performed to compare the subgroups of lenalidomide plus dexamethasone-treated patients (i.e. those who had received one prior therapy vs. those who had received two or more therapies) in terms of efficacy outcome, as well as the baseline characteristics.

### Baseline characteristics

Of 353 patients who received lenalidomide plus dexamethasone, 133 had received one prior therapy before this combination therapy, and 220 had received two or more prior therapies. Significant baseline differences in those patients who had received one prior therapy vs. those with two or more prior therapies include: prior ASCT (66.9% vs. 53.2%; *P*=0.014); prior treatment with thalidomide (9.8% vs. 51.8%; *P*<0.001); and prior treatment with bortezomib (1.5% vs. 11.4%; *P*<0.001) (Table 2). The average length of time from diagnosis was also significantly different between patients with one prior therapy and those who had received two or more prior therapies (2.2 yr vs. 4.1 yr; *P*<0.001).

**Table 2:** Baseline characteristics and treatment history of patients according to number of prior therapies

	Lenalidomide plus dexamethasone		
	1 prior therapy (*n*=133)	≥2 prior therapies (*n*=220)	*P*-value
Median age, yr	62.1	63.1	0.34
Male sex, *n* (%)	82 (61.7)	128 (58.2)	0.58
Baseline β_2_-microglobulin ≤2.5 mg/L, *n* (%)	47 (35.3)	56 (25.5)	0.054
Baseline β_2_-microglobulin >2.5 mg/L, *n* (%)	86 (64.7)	164 (74.5)	0.054
ECOG score 0–1, *n* (%)	119 (89.5)	188 (85.5)	0.77
Median time from diagnosis, yr (range)	2.2 (0.4–9.7)	4.1 (0.5–15.7)	<0.001
Prior ASCT, *n* (%)	89 (66.9)	117 (53.2)	0.014
Prior treatment with thalidomide, *n* (%)	13 (9.8)	114 (51.8)	<0.001
Prior treatment with bortezomib, *n* (%)	2 (1.5)	25 (11.4)	<0.001

ASCT, autologous stem cell transplantation; ECOG, Eastern Cooperative Oncology Group.

### Treatment

Patients with one prior therapy had a median treatment duration of 12.5 months (range: 0.3–24.1) which was higher than that for patients with two or more prior therapies (9.2 months, range: 0.03–24.8; *P*<0.001) (Table 3).

**Table 3:** Outcomes in patients by number of prior therapies

	Lenalidomide plus dexamethasone		
	1 prior therapy (*n*=133)	≥2 prior therapies (*n*=220)	*P*-value
Response rates, *n* (%)			
Overall response	89 (66.9)	125 (56.8)	0.060
CR	27 (20.3)	26 (11.8)	0.028
VGPR	26 (19.5)	35 (15.9)	
CR + VGPR	53 (39.8)	61 (27.7)	0.025
Partial response	36 (27.1)	64 (29.1)	
Stable disease	30 (22.6)	77 (35.0)	
Progressive disease	6 (4.5)	2 (0.9)	
Response not evaluable	8 (6.0)	16 (7.3)	
Median duration of treatment, months (range)	12.5 (0.3–24.1)	9.2 (0.03–24.8)	<0.001
Median duration of response, months (range)	NR (11.4–NR)	13.0 (8.4–NR)	0.21
Patients who relapsed, %	34.5	44.4	0.16
Patients who had a dose reduction^1^, %	33.1	38.0	0.36
Patients who discontinued due to toxicity, %	14.3	14.5	0.54

1 With or without interruption in lenalidomide treatment.

NR, not reached; CR, complete response; VGPR, very good partial response.

The proportion of patients who had a dose reduction, with or without interruption of lenalidomide treatment, was similar among those who had undergone either one or at least two prior therapies (33.1% vs. 38.0%; *P*=0.36). There was also no significant difference in discontinuation rates due to toxicity between these patients (14.3% vs. 14.5%; *P*=0.54).

### Response

Patients who had received one prior therapy had a trend towards a higher ORR than those receiving at least two prior therapies (66.9% vs. 56.8%; *P*=0.06) (Table 3). However, more patients who had one prior therapy achieved a complete response or a very good partial response (CR + VGPR; 39.8%) compared with those who had two or more prior therapies (27.7%; *P*=0.025). With a median follow-up of 15.5 months for responders, the median duration of response had not been reached in the cohort who had received only one prior therapy, whereas those patients with two or more prior therapies had a median response duration of 13.0 months (*P*=0.21). Fewer responders receiving one prior therapy (38.2%) had relapsed compared with those receiving two or more therapies (45.6%) at the time of study unblinding.

**Figure 1:**
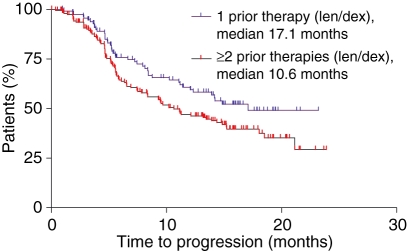
Kaplan–Meier estimates of the time to progression of patients who received lenalidomide plus dexamethasone (len/dex) by number of prior therapies (*P*=0.026), using data up to unblinding.

**Figure 2:**
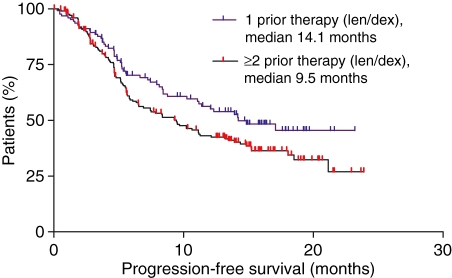
Kaplan–Meier estimates of progression-free survival in the lenalidomide plus dexamethasone (len/dex) group by number of prior therapies (*P*=0.047), using data up to unblinding.

TTP was significantly longer for patients who had received one prior therapy compared with those who had at least two prior therapies (17.1 months vs. 10.6 months; hazard ratio (HR) 0.68; 95% confidence interval (CI) 0.48–0.97; *P*=0.026) (Fig. 1). PFS was also significantly longer in patients who had received one prior therapy compared with those who had received two or more prior lines of therapy (median of 14.1 months vs. 9.5 months; HR 0.71; 95% CI 0.2–0.99; *P*=0.047) (Fig. 2).

**Figure 3:**
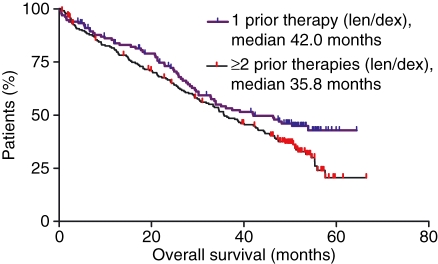
Kaplan–Meier estimates of overall survival in the lenalidomide plus dexamethasone (len/dex) group by number of prior therapies (*P*=0.041), using updated data as of December 2008.

OS from study enrollment was significantly longer in patients who had received one prior therapy than in those who had received at least two prior lines of therapy at the time of unblinding (median not reached vs. 30.8 months, HR 0.59; 95% CI 0.36–0.95; *P*=0.028). Importantly, this significant survival advantage was sustained with the extended follow-up based on data as of December 2008 (median OS of 42.0 vs. 35.8 months, *P*=0.041) (Fig. 3).

### Safety

NCI-CTC grade 3 and 4 neutropenia was the most common adverse event associated with lenalidomide plus dexamethasone treatment in patients with one prior therapy, and in those with at least two prior therapies (41.4% vs. 31.8%) (Table 4). There was no difference in the incidence of febrile neutropenia between patients with one prior therapy and those with two or more prior therapies (1.6% vs. 2.6%; *P*=0.72). Thrombotic events, including deep-vein thrombosis and pulmonary embolism, were the most common grade 3 and 4 non-hematologic toxicities, occurring in 10.5% of patients with one prior therapy vs. 12.3% of those with at least two prior therapies (*P*=0.63). No grade 3 and 4 peripheral neuropathy was observed in patients treated with lenalidomide plus dexamethasone with one prior therapy, whereas it was reported in 2.3% of the patients with two or more prior therapies.

**Table 4:** Incidence of National Cancer Institute Common Toxicity Criteria (NCI-CTC) grade 3 and 4 adverse events

	Lenalidomide plus dexamethasone	
	1 prior therapy (*n*=133)	≥2 prior therapies (*n*=220)
Hematologic toxicities, *n* (%)		
Anemia	13 (9.8)	25 (11.4)
Thrombocytopenia	12 (9.0)	34 (15.5)
Neutropenia	55 (41.4)	70 (31.8)
Infection	24 (18.0)	29 (13.2)
Febrile neutropenia	2 (1.6)	6 (2.6)
Non-hematologic toxicities, *n* (%)		
Deep-vein thrombosis/pulmonary embolism	14 (10.5)	27 (12.3)
Peripheral neuropathy	0 (0.0)	5 (2.3)
Fatigue	10 (7.5)	13 (5.9)
GI (nausea, vomiting, constipation)	8 (6.0)	7 (3.2)

GI, gastrointestinal.

## Discussion

The results of this analysis of pooled data from the two phase III studies MM-009 and MM-010 assessing lenalidomide plus dexamethasone showed that patients with fewer prior treatments will benefit more from this active combination. The number of lines of treatment had a greater impact than the type of prior therapy (e.g. prior thalidomide or bortezomib treatment). The ORR after receiving lenalidomide plus dexamethasone was higher in patients receiving lenalidomide plus dexamethasone after only one prior therapy compared with those with two or more prior therapies, although the difference was not statistically significant. These overall results are consistent with the results previously reported by Wang *et al.* for the subset of patients who receive prior thalidomide (14). The quality of response was significantly better in patients receiving lenalidomide plus dexamethasone after only one prior therapy, as shown by the statistically higher CR and VGPR rates in these patients. In addition, duration of response was longer in patients with one prior therapy compared with those with two or more prior therapies. The median TTP reached in this study for patients treated in the second-line setting was 17.1 months. This significant benefit was diminished if lenalidomide plus dexamethasone treatment was given later in treatment. The median OS at 42 months was also significantly longer for those with only one prior therapy than for those with two or more prior therapies and is among the longest reported in the literature to date for these patients (2–11).

The incidence of NCI-CTC grade 3 and 4 adverse events was similar for patients who had had either one or at least two prior therapies, with neutropenia occurring most frequently. The incidence of thrombotic events was comparable between the two groups. Overall, treatment with lenalidomide plus dexamethasone was well tolerated, with a significantly longer treatment duration for first relapse compared to later lines of therapy. This longer treatment duration in the second line did not generally increase toxicity, rate of dose reduction, or treatment discontinuation compared to later lines of therapy with shorter treatment duration. For those patients with one or two or more prior therapies, the incidence of treatment-emergent peripheral neuropathy was low. It is interesting to note that despite being a more heavily pretreated group, those with two or more prior therapies generally did not experience more treatment-emergent adverse events.

The patients in this study with one prior therapy were not only less heavily pretreated, they were also fewer years from diagnosis than those with two or more prior therapies. This could account for potential differences in the biology of the disease and could likely explain, in part, the better outcomes observed for patients in first relapse. However, β_2_-microglobulin, a typical strong predictor of patient outcomes and indicator of advanced disease, was not statistically different in these two patient subsets. It must also be noted that these results were achieved at a time when novel agents were rarely used upfront, and results might be different in patients who received novel agents as first-line treatment.

Results of this overall analysis of first relapse from the MM-009/MM-010 phase III studies confirmed those previously reported by Wang *et al.* specifically for lenalidomide plus dexamethasone after thalidomide treatment (14). Wang reported that lenalidomide plus dexamethasone received as second-line therapy immediately after thalidomide treatment, resulted in a higher ORR and longer median TTP than when used later in the treatment, after other additional therapies. This further supports the significant role of lenalidomide plus dexamethasone as second-line treatment, regardless of prior exposure to thalidomide.

In conclusion, lenalidomide plus dexamethasone treatment resulted in significantly prolonged TTP, PFS, and OS, as well as better quality of response, when used at first relapse compared with its use later as salvage therapy. Lenalidomide plus dexamethasone should be considered as a second-line therapy for patients with MM. Trials using lenalidomide plus dexamethasone are also underway to confirm benefits earlier in MM treatment as part of first-line therapy.
